# Autophagy is increased following either pharmacological or genetic silencing of mGluR5 signaling in Alzheimer’s disease mouse models

**DOI:** 10.1186/s13041-018-0364-9

**Published:** 2018-04-10

**Authors:** Khaled S. Abd-Elrahman, Alison Hamilton, Maryam Vasefi, Stephen S. G. Ferguson

**Affiliations:** 1 University of Ottawa Brain and Mind Institute, University of Ottawa, 451 Smyth Road, Ottawa, ON K1H 8M5 Canada; 2 Department of Cellular and Molecular Medicine, University of Ottawa, 451 Smyth Road, Ottawa, ON K1H 8M5 Canada; 3 Department of Pharmacology and Toxicology, Faculty of Pharmacy, University of Alexandria, Alexandria, 21521 Egypt; 4 Lamar University, Beaumont, TX 77710 USA

**Keywords:** GPCR, mGluR5, Alzheimer’s disease, APPswe/PS1ΔE9, 3xTg-AD, CTEP, ULK1, ZBTB16, Autophagy

## Abstract

Alzheimer’s disease (AD) is characterized by neurotoxicity mediated by the accumulation of beta amyloid (Aβ) oligomers, causing neuronal loss and progressive cognitive decline. Genetic deletion or chronic pharmacological inhibition of mGluR5 by the negative allosteric modulator CTEP, rescues cognitive function and reduces Aβ aggregation in both APPswe/PS1ΔE9 and 3xTg-AD mouse models of AD. In late onset neurodegenerative diseases, such as AD, defects arise at different stages of the autophagy pathway. Here, we show that mGluR5 cell surface expression is elevated in APPswe/PS1ΔE9 and 3xTg-AD mice. This is accompanied by reduced autophagy (accumulation of p62) as the consequence of increased ZBTB16 expression and reduced ULK1 activity, as we have previously observed in Huntington’s disease (HD). The chronic (12 week) inhibition of mGluR5 with CTEP in APPswe/PS1ΔE9 and 3xTg-AD mice prevents the observed increase in mGluR5 surface expression. In addition, mGluR5 inactivation facilitates the loss of ZBTB16 expression and ULK1 activation as a consequence of ULK-Ser757 dephosphorylation, which promotes the loss of expression of the autophagy marker p62. Moreover, the genetic ablation of mGluR5 in APPswe/PS1ΔE9 mice activated autophagy via similar mechanisms to pharmacological blockade. This study provides further evidence that mGluR5 overactivation contributes to inhibition of autophagy and can result in impaired clearance of neurotoxic aggregates in multiple neurodegenerative diseases. Thus, it provides additional support for the potential of mGluR5 inhibition as a general therapeutic strategy for neurodegenerative diseases such as AD and HD.

## Introduction

Alzheimer’s disease (AD) is the most prevalent of all the neurodegenerative diseases, with an alarming rise in prevalence as a result of an aging population [1]. AD presents as progressive memory loss and cognitive decline and current therapeutic strategies are not curative with limited efficacy [2, 3]. Beta-amyloid (Aβ) protein, a product of amyloid precursor proteins (APP) cleavage that forms soluble oligomers and fibrillar plaques, is considered the principal neurotoxic species in AD brains along with neurofibirillary tangles comprised of phosphorylated Tau protein [4–6].

Metabotropic glutamate receptor 5 (mGluR5) is a member of the G protein-coupled receptor (GPCR) superfamily and when activated by glutamate couples to the heterotrimeric G protein Gα_q/11_ [7]. mGluR5 also functions as an extracellular scaffold for Aβ oligomers. Activation of mGluR5 by Aβ oligomers leads to the release of Ca^2+^ from intracellular stores and a consequent disruption in synaptic signaling and function [8–10]. We have shown that the genetic deletion of mGluR5 in the APPswe/PS1ΔE9 (APPswe) mouse model of AD improved cognitive function and reduced AD pathogenesis [11]. Moreover, the pharmacological blockade of mGluR5 using the mGluR5-selective negative allosteric modulator CTEP, reduces the development of AD-like neuropathology; specifically reducing Aβ soluble oligomer and plaque deposition, in both APPswe/PS1ΔE9 and 3xTg-AD (3xTg) mouse models [12]. Similarly, mGluR5 knockout and pharmacological blockade results in delayed disease progression and a reduction in huntingtin pathology in preclinical mouse models of Huntington’s disease (HD) which we have linked to increased autophagy via alterations in both Zinc finger and BTB domain-containing protein 16 (ZBTB16)- and Unc-51-like kinase 1 (ULK1)-dependent mechanisms [13, 14]. Specifically, we showed that pharmacological blockade of mGluR5 in HD mice reduced the expression of ZBTB16, key component of the ZBTB16-Cullin3-Roc1 E3-ubiquitin ligase complex, leading to rescue of the key autophagy adaptor ATG14. mGluR5 inhibition also activated ULK1 that was essential for phosphorylation of the autophagy factor ATG13, required for autophagosome formation [14, 15]. However, it remains unclear whether alterations in these autophagy pathways due to aberrant mGluR5 signaling are also evident in mouse models of AD.

Here, we show that the reduction in Aβ burden and improvement in memory function following mGluR5 pharmacological antagonism or genetic knockout in APPswe mice [11, 12], is paralleled by a reduction of increased cell surface mGluR5 expression in APPswe and 3xTg mice as well as the reduction of the autophagy marker p62 as the consequence of reduced ZBTB16 expression and increased ULK1 activity. These findings using two different approaches to silence mGluR5 as well as two different mouse models of AD further support the pivotal role of mGluR5 in AD pathogenesis.

## Results

### Chronic mGluR5 antagonism reduces mGluR5 cell surface expression in AD mouse models

**Fig. 1:**
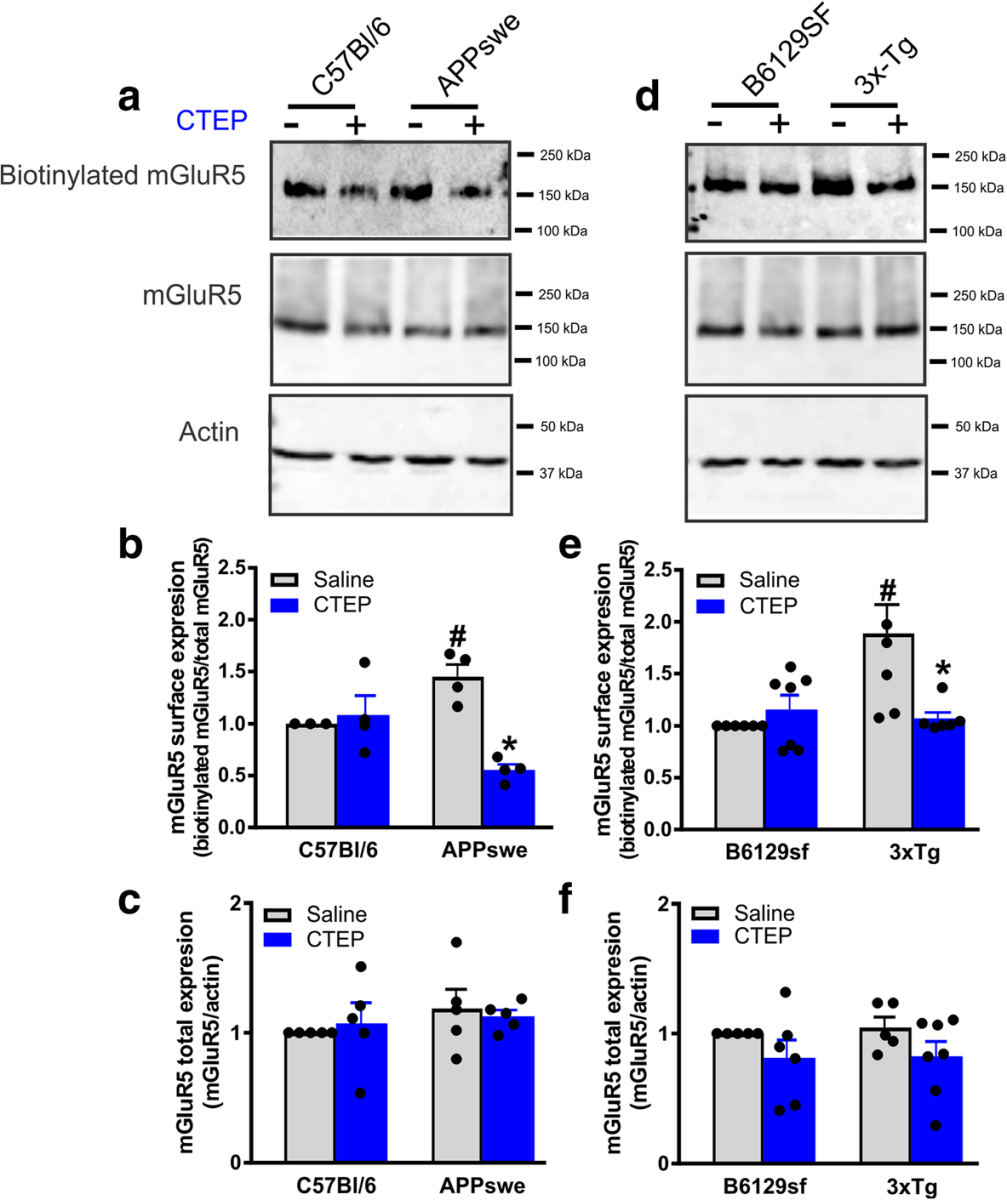
CTEP reduces surface but not total expression of mGluR5 in AD mice. **a** Representative western blots and mean ± SEM of mGluR5 surface expression **b** and total expression **c** in brain lysates from APPswe/PS1∆E9 (APPswe) and C57Bl/6 controls mice after chronic treatment with either saline or CTEP (2 mg/kg). **d** Representative western blots and mean ± SEM of mGluR5 surface expression **e** and total expression **f** in brain lysates from 3xTg-AD (3xTg) and B6129sf control mice after chronic treatment with either saline or CTEP. Values are expressed as a fraction of the saline-treated control. Surface expression represents quantification of biotinylated mGluR5 relative to total mGluR5 expression. Total mGluR5 expression was normalized to actin (*n* = 4–6 for each group). # Significantly different (*P* < 0.05) from corresponding C57B6/l or B6129sf values, * significantly different (*P* < 0.05) from saline-treated value

Aβ oligomers were previously reported to activate mGluR5 and trigger their clustering, thereby contributing to the glutamate excitotoxicity at the neuronal synapses in the AD brain [8–10]. We have also reported an elevated mGluR5 cell surface expression in 12-month-old APPswe mice. Here, we tested whether elevated mGluR5 cell surface expression contribute to AD pathology in another AD model, the 3xTg mouse model. We also examined whether chronic (12-week) inhibition of mGluR5 using CTEP (2 mg/kg) in 9-month-old APPswe and 3xTg mice could normalize mGluR5 cell surface expression and, thereby might contribute to the favorable outcome of mGluR5 inhibition on cognitive function in both mouse models. The highly potent mGluR5-speciific negative allosteric modulator CTEP (2-chloro-4-[2[2,5-dimethyl-1-[4-(trifluoromethoxy) phenyl] imidazol-4-yl] ethynyl] pyridine) was chosen for this study because it is orally bioavailable, crosses the blood brain barrier, has a half-life of 18 h and its analogue, Basimglurant, was proven to be well- tolerated in phase II trials for major depressive disorder [16, 17]. Coronal brain slices from 12-month-old APPswe, 3xTg and control mice (C59Bl/6 for APPswe and B6129sf for 3xTg) after 12 weeks of intraperitoneal injection with either CTEP or saline were employed in a cell surface biotinylation assay to determine total and cell membrane expression of mGluR5. We detected an increase in the cell surface expression of mGluR5 in the brain slices from saline-treated APPswe and 3xTg brains when compared to wild-type mice (Fig. 1a, b, d and e). Interestingly, the increase in mGluR5 surface expression was reversed in CTEP-treated AD mice and the values were indistinguishable from controls. No significant change in the total expression of mGluR5 was detected in all groups (Fig. 1c and f). Taken together, these results indicated that chronic antagonism of mGluR5 with a selective negative allosteric modulator (NAM) could block the increase in cell surface expression of mGluR5 and thus may contribute to slowing the progression AD pathology and improvement in cognitive function.

### Pharmacological and genetic silencing of mGluR5 activate autophagy via a ZBTB16-regulated pathway

**Fig. 2:**
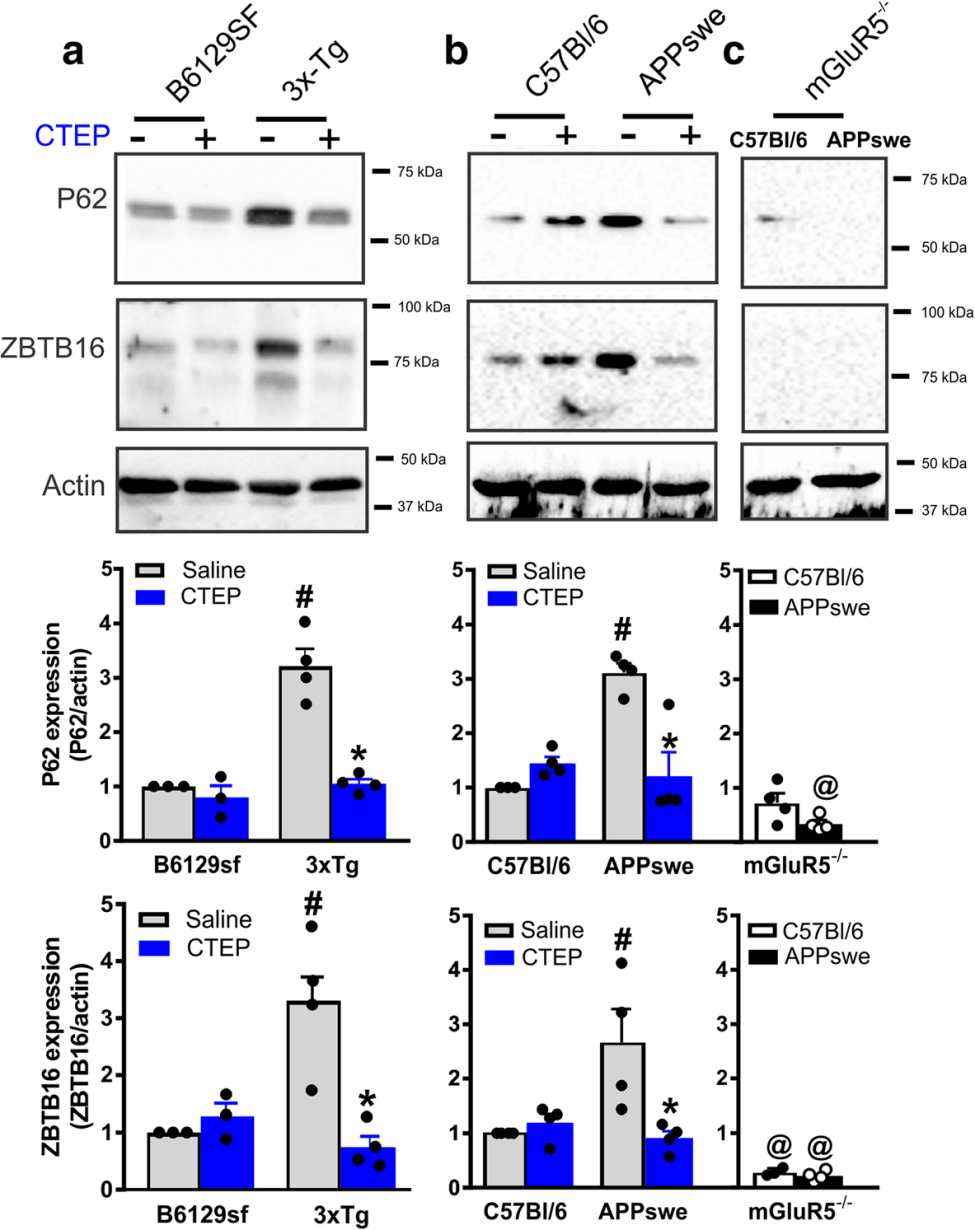
ZBTB16-mediated activation of autophagy in AD mice is mGluR5-dependent. **a** Representative western blots and mean ± SEM of P62 and ZBTB16 in brain lysates from 3xTg-AD (3xTg) and control B6129sf mice after chronic treatment with either saline or CTEP (2 mg/kg). **b** Representative western blots and mean ± SEM of P62 and ZBTB16 in brain lysates from APPswe/PS1∆E9 (APPswe) and control C56Bl/6 mice after chronic treatment with either saline or CTEP or **c** genetic deletion of mGluR5 (mGluR5^−/−^). Representative Blots in panel from B and C are from the same blot. Values are expressed as a fraction of the saline-treated control. ZBTB16, and P62 are normalized to actin (*n* = 4 for each group). # Significantly different (*P* < 0.05) from corresponding control for each AD mouse model, * significantly different (*P* < 0.05) from saline-treated value. @ significantly different (*P* < 0.05) from saline treated C57Bl/6 or APPswe

Zhang and colleagues reported a novel pathway through which GPCR signaling inhibits autophagy. Specifically, GPCR activation stabilized the expression of the transcription factor ZBTB16, a key component of the ZBTB16-Cullin3-Roc1 E3-ubiquitin ligase, which promoted the degradation of various autophagy adaptor proteins to inhibit autophagy [18]. Therefore, we wanted to determine whether silencing mGluR5 signaling either pharmacologically or genetically would reduce ZBTB16 expression in 3xTg and APPswe mice to activate autophagy. We examined brain lysates derived from 12-month old mice APPswe after 12-week treatment with either CTEP or saline as well as age-matched APPswe lacking mGluR5 (APPswe/mGluR5^−/−^). We found that the expression of the ubiquitin ligase component ZBTB16 and the autophagy marker p62 were increased in both saline-treated 3xTg and APPswe mice and that chronic treatment of these mice with CTEP for 12 weeks reduced both ZBTB16 and p62 protein expression to values comparable to wild-type control mice (Fig. 2a and b). We also detected a reduction in p62 and ZBTB16 expression in APPswe/mGluR5^−/−^ and significantly lower levels of ZBTB16 in mGluR5^−/−^ compared to wild-type C57Bl/6 mice (Fig. 2c). Taken together, these data indicate an obligatory role of mGluR5 in ZBTB16-regulated activation of autophagy.

### Pharmacological and genetic silencing of mGluR5 activates ULK1

**Fig. 3:**
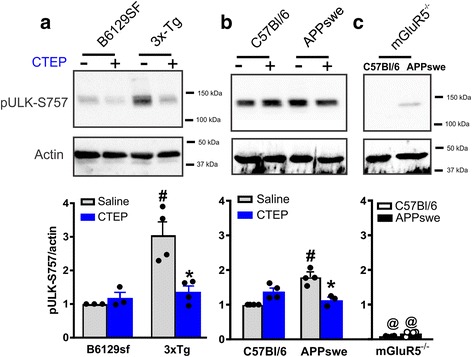
ULK1 activation plays a role in mGluR5-dependent activation of autophagy in AD mice. **a** Representative western blots and mean ± SEM of pULK-Ser757 in brain lysates from 3xTg-AD (3xTg) and control B6129sf mice after chronic treatment with either saline or CTEP (2 mg/kg). **b** Representative western blots and mean ± SEM of pULK-Ser757 in brain lysates from APPswe/PS1∆E9 (APPswe) and control C56Bl/6 mice after chronic treatment with either saline or CTEP or **c** genetic deletion of mGluR5 (mGluR5^−/−^). Representative Blots in panel from B and C are from the same blot. Values are expressed as a fraction of the saline-treated control. pULK-Ser757 is normalized to actin (*n* = 4 for each group). # Significantly different (*P* < 0.05) from corresponding control for each AD mouse model, * significantly different (*P* < 0.05) from saline-treated value. @ significantly different (*P* < 0.05) from saline treated C57Bl/6 or APPswe

ULK family members are ubiquitously expressed kinases that localize to the phagophore membrane upon nutrient starvation to promote autophagosome formation [19, 20]. mTOR phosphorylates ULK1 at Ser757 suppressing its kinase activity and autophagy initiation [15]. mGluR5 is known to activate the mTOR pathway and it reduced ULK1-dependent activation of autophagy in zQ175 HD mice [14, 21] . Here, we tested whether blocking mGluR5 could induce autophagy by activating ULK1 dephosphorylation in 3xTg and APPswe mouse models of AD. Chronic blockade of mGluR5 with CTEP reduced the inhibitory phosphorylation of ULK1 at Ser757 observed in saline-treated 3xTg and APPswe mice (Fig. 3a and b) to values comparable to corresponding wild-type levels indicating an increase in ULK1 activity following CTEP treatment. Interestingly, genetic deletion of mGluR5 completely abolished ULK1-Ser757 phosphorylation in wild-type and APPswe mice (Fig. 3c). Together, these findings strongly support the role of ULK1 in mGluR5-mediated regulation of autophagy and that mGluR5 can potentially alter autophagy via multiple convergent mechanisms and this correlates with the clearance of proteotoxic aggregates, in this case β-amyloid plaques.

## Discussion

mGluR5 antagonism using highly-selective mGluR5 NAMs represents a promising approach to slow disease progression and proteotoxic protein aggregation in both AD and HD [12, 14]. In the current study, we provide further evidence for the pivotal role of mGluR5 in AD pathogenesis by demonstrating an increase in mGluR5 surface expression in two AD mouse models, the APPswe and 3xTg models. We also show for, the first time, that the mGluR5 NAM CTEP could abolish the increase in membrane delivery of mGluR5 that is correlated with an activation of autophagy via a ZBTB16- and ULK1- dependent pathways in both mouse models of AD. Moreover, the genetic deletion of mGluR5 in APPswe resulted in similar outcomes to the pharmacological inhibition with a mGluR5 NAM and further supports the role of mGluR5-mediated regulation of autophagy in the pathology of AD. The observation that this molecular pathway is similarly activated in a zQ175 mouse model of HD following treatment with the mGluR5 NAM, CTEP [14], indicates that mGluR5 contributes neurodegenerative disease processes via a conserved mechanism.

This study extends our previous work using both genetic and pharmacological approaches to implicate mGluR5 in the pathophysiological hallmarks of AD. We reported that the pharmacological inhibition and genetic ablation of mGluR5 corrected spatial memory loss and reduced formation of Aβ oligomers and plaques in AD mice [11, 12]. Here, we provide further evidence for the role of mGluR5 signaling in AD by demonstrating an increase in the delivery of mGluR5 to the plasma membrane in brain slices derived from both APPswe and 3xTg mice mouse models of AD. The increase in mGluR5 surface expression is particularly interesting as Aβ oligomers interact with mGluR5 and potentially functions to accelerate Aβ production via the mGluR5- and Fragile X mental retardation protein (FMRP)-dependent activation of the amyloidogenic pathway [10, 11]. These Aβ oligomers also trigger the clustering and activation of mGluR5 resulting in the release of Ca^2+^ from intracellular stores, an event that is critical for the neurotoxic signaling at glutamatergic synapses [8, 9]. The enhanced delivery of mGluR5 means that the receptor is more readily accessible for activation and clustering by Aβ oligomers, thereby potentially accelerating glutamatergic excitotoxicity, synaptic loss and AD progression. Thus, CTEP via its allosteric binding to mGluR5 may function to disrupt a proposed Aβ oligomer-mediated neurodegenerative positive feedback loop. The disruption of this feedback loop may contribute in part to a reduction in the formation of Aβ oligomers and plaques resulting in improvement in memory and cognitive function. Our findings support a critical/central contribution of pathological mGluR5 signaling to the pathophysiology underlying AD.

Studies using transgenic mice with impaired central nervous system autophagy reported an increase in ubiquitinated protein inclusions and neurodegeneration [22, 23]. This observation strongly suggests an obligatory role of autophagy in neuronal health. Autophagy is a catabolic process that clears cellular organelles and protein aggregates and defects in autophagy have been increasingly implicated in proteinopathies such as AD, HD and Parkinson’s disease [24–27]. We have demonstrated that mGluR5 signals through a ZBTB16-Cullin3-Roc1 E3-ubiquitin ligase pathway to inhibit autophagy in a zQ175 HD mouse model, which we have correlated with the accumulation of mutant huntingtin aggregates and disease progression. mGluR5 blockade with CTEP triggers the degradation of ZBTB16 to rescue autophagy adaptor protein ATG14 and activates autophagy that is associated with a reduction in mutant huntingtin aggregation. Moreover, mGluR5 blockade reduces ULK1-Ser757 phosphorylation and to allow autophagy adaptor-initiated autophagosome biogenesis [14]. Similar to HD mice, in the study we detect a significant elevation in ZBTB16 and p62 expression, as well as ULK1-Ser757 phosphorylation in both APPswe and 3X-Tg mice that is significantly attenuated following CTEP treatment. In addition, we also employ an alternative approach to knockout mGluR5 in APPswe via the genetic deletion of mGluR5. This resulted in an even more robust reduction in ZBTB16, P62 and pULK1-Ser757 phosphorylation levels compared to the pharmacological approach. These findings along with our previous reports showing a reduction Aβ oligomers and plaques [11, 12] indicates that mGluR5 signaling via ZBTB16 and ULK1 is crucial to maintain adequate clearance of these proteotoxic aggregates. Thus, alterations in mGluR5-mediated signaling in the brain is expected to accelerate the process of neurodegeneration in AD.

In summary, we find that mGluR5 antagonism represents an effective approach to slow and potentially reverse AD progression both at the receptor level, by reducing mGluR5 membrane trafficking, and at the signaling level, by activating autophagy. Moreover, findings from this study and our previous work in zQ175 HD mice [14] provide evidence to support a conserved mechanism of autophagy inhibition downstream of mGluR5 that potentially reduces the clearance of toxic misfolded protein species in both AD and HD. This study also extends on our previous observations that pharmacologically targeting a single GPCR may be effective in clearing neurotoxic aggregates through autophagy via a well-tolerated novel therapeutic approach. These findings warrant further investigation of the role of ZBTB16- and mTOR-mediated autophagic pathway in other neurodevelopmental and neurodegenerative diseases in which mGluR5 has been previously implicated [28, 29]. This will provide a better understanding of the common pathophysiological signals in neurodegeneration and ultimately novel therapeutic approaches to target these aberrant signals.

## Materials and methods

### Reagents

CTEP was purchased from Axon Medchem. Horseradish peroxidase (HRP)-conjugated anti-rabbit IgG secondary antibody was from Bio-Rad(1662408EDU). Rabbit anti-actin (CL2810AP) was from Cedarlane (Burlington, Ontario). Mouse anti-P62 (56416) and rabbit anti-ZBTB16 (39354) antibodies were from Abcam (Cambridge, Massachusetts). Rabbit anti-mGluR5 antibody (AB5675) was from Millipore (Billerica, Massachusetts). Anti-phospho ULK1-Ser^757^ (14202) from Cell Signaling Technology (Danvers, Massachusetts). Reagents used for western blotting were purchased from (Bio-Rad Laboratories, Hercules, California) and all other biochemical reagents were from Sigma-Aldrich (St. Louis, Missouri).

### Animals

STOCK B6C3-Tg (APPswe/PSEN1ΔE9)85Dbo/J mice that carry the human APP with Swedish mutation and the DeltaE9 mutation of the human presenilin 1 gene [30], mGluR5 knockout mice B6;129-Grm5tm1Rod/J (mGluR5^−/−^) [31] and 3xTg-AD mice that carry both the human APP with Swedish mutation and the DeltaE9 mutation of the human presenilin 1 gene along with a tau P301L mutation [32] were purchased from Jackson Laboratory (Bar Harbor, ME). APPswe/PS1ΔE9/mGluR5^−/−^ mice were generated by crossing APPswe/PS1ΔE9 mice with a C57/Bl6 background, with mGluR5^−/−^ C57Bl/6 females. Offspring were tail snipped and genotyped using PCR with primers specific for the APP sequence and primers specific for mGluR5. Animals were housed in an animal care facility in cages of 2 or more animals, received food and water ad libitum and were maintained on a 12-h light/12 h dark cycle at 24 °C. Two sets of male APPswe/PSEN1ΔE9, 3xTg-AD mice and their wild-type control mice (C57Bl/6 control for APPswe and B6129sf control for 3xTg) were aged to 9 months and were then treated by intraperitoneal injection with a 200 μl volume of either vehicle (saline control) or CTEP (2 mg/kg, final concentration 20 nM) every 48 h for 12 weeks by a blinded technician. At the end of the 12-week treatment, mice were sacrificed by exsanguination and brains were collected and randomized for biochemical determinations. mGluR5^−/−^ and APPswe/PS1ΔE9/mGluR5^−/−^ mice were aged to 12 months and sacrificed by exsanguination and brains were collected for biochemical determinations.

### Cell surface biotinylation

Cell surface biotinylation was performed as previously described [7, 11]. Coronal brain slices (350 μm) from saline- or CTEP-treated wild-type, APPswe and 3xTg mice were prepared using a vibratome system (Leica). Slices were recovered in KREBS buffer (127 mM NaCl, 2 mM KCl, 10 mM glucose, 1.2 mM KH_2_PO_4_, 26 mM NaH_2_CO_3_, 1 mM MgSO_4_, 1 mM CaCl_2_, pH 7.4) continuously gassed with 95%O_2_/5%CO_2_ for 30 min at 37 °C. Slices were transferred to tubes and biotinylated for 1 h in 1.5 mg/ml sulfo-NHS-SS-biotin on ice. Slices were then washed and biotinylation was quenched with 100 μM glycine in HBSS for 30 min on ice. Following washes in HBSS, tissue was lysed in RIPA buffer (0.15 M NaCl, 0.05 M Tris–HCl, pH 7.2, 0.05 M EDTA, 1% Nonidet P40, 1% Triton X-100, 0.5% sodium deoxycholate, 0.1% SDS) containing protease inhibitors (1 mM AEBSF, 10 μg/ml leupeptin, and 2.5 μg/ml aprotinin). Biotinylated proteins were then precipitated with NeutrAvidin beads using equivalent amounts of proteins for each sample. Biotinylated proteins were subjected to SDS-polyacrylamide gel (SDS-PAGE) and immunoblotted with Rabbit polyclonal mGluR5 antibody (1:1000, dilution), as described below.

### Immunoblotting

Brain hemispheres was lysed in 1.5 ml ice-cold lysis buffer (50 mM Tris, pH 8.0, 150 mM NaCl, and 1% Triton X-100) containing protease inhibitors (1 mM AEBSF, 10 μg/ml leupeptin, and 2.5 μg/ml aprotinin) and phosphatase inhibitors (10 mM NaF and 500 μM Na_3_VO_4_) and centrifuged at 15000 rpm at 4 °C for 15 min. The supernatant was collected and total protein levels were quantified using Bradford Protein Assay (Bio-Rad). Homogenates were diluted in a mix of lysis buffer and β-mercaptoethanol containing 3× loading buffer and boiled for 10 min at 95 °C. Aliquots containing 25 μg total proteins were resolved by electrophoresis on a 7.5% SDS-PAGE and transferred onto nitrocellulose membranes. Blots were blocked in Tris-buffered saline, pH 7.6 containing 0.05% of Tween 20 (TBST) and 5% non-fat dry milk for 2 h at room temperature and then incubated overnight at 4 °C with primary antibodies diluted 1:1000 in TBST containing 1% non-fat dry milk. Immunodetection was performed by incubating with secondary antibodies (anti-rabbit/mouse) diluted 1:5000 in TBST containing 1% of non-fat dry milk for 1 h. Membranes were washed in TBST and then bands were detected and quantified using BioRad chemiluminescence system.

### Statistical analysis

Means ± SEM are shown for each of independent experiments are shown in the various figure legends. GraphPad Prism software was used to analyze data for statistical significance. Statistical significance was determined by a series of 2 (strain) × 2 (drug treatment) ANOVAs followed by Fisher’s LSD comparisons for the significant main effects or interactions.

## Abbreviations

3xTg: 3xTg-AD
AD: Alzheimer’s disease
APP: Amyloid precursor protein
APPswe: APPswe/PS1ΔE9
APPswe/ mGluR5^−/−^: APPswe/PS1ΔE9 lacking mGluR5
ATG14: Autophagy-related protein 14
Aβ: Amyloid beta
CTEP: 2-chloro-4-[2[2,5-dimethyl-1-[4-(trifluoromethoxy) phenyl] imidazol-4-yl] ethynyl] pyridine
FMRP: Fragile X mental retardation protein
GPCR: G protein-coupled receptor
HD: Huntington’s disease
mGluR5: metabotropic glutamate receptor 5
mTOR: mammalian target of rapamycin
NAM: Negative allosteric modulator
ULK: Unc-51-like kinase
ZBTB16: Zinc finger and BTB domain-containing protein 16
